# SG-APSIC1158: COVID-19 vaccine booster hesitancy among healthcare workers: A retrospective observational study in Singapore

**DOI:** 10.1017/ash.2023.12

**Published:** 2023-03-16

**Authors:** Sky Wei Chee Koh, Hwei Ming Tan, Wayne Han Lee, Jancy Mathews, Doris Young

**Affiliations:** National University Health System, Singapore; Singapore National University Health System, Singapore; Singapore National University Health System, Singapore; Singapore National University Health System, Singapore; Singapore National University Health System, Singapore

## Abstract

**Objectives:** COVID-19 booster uptake has remained poor among healthcare workers (HCWs) despite evidence of improved immunity against the SARS-COV-2 δ (delta) and ο (omicron) variants. Although most studies have used a questionnaire to assess hesitancy, we aimed to identify factors affecting booster hesitancy by examining actual vaccine uptake across time. **Methods:** COVID-19 vaccination database records were extracted for HCWs working at 7 Singaporean public primary-care clinics between January and December 2021. Data included sex, profession, place of practice, vaccination type, and dates. Time to booster was calculated from the date of vaccination minus the date of eligibility. The χ2 test was used to compare the relationship between first dose and booster hesitancy. The Kaplan-Meier method and the log-rank test were used to evaluate differences in cumulative booster uptake. Multivariate Cox regression was used to investigate predictors of timely booster vaccination. The vaccination rate was charted across time and was corroborated with media releases pertaining to legislative changes. **Results:** Of 891 primary-care HCWs, 877 (98.9%) were fully vaccinated and 73.8% of eligible HCWs had taken the booster. HCWs were less booster hesitant (median, 16 days; range, 5–31.3) compared to the first dose (median, 39 days; range, 13–119.3). First-dose–hesitant HCWs were more likely to be booster hesitant (OR, 3.66; 95% CI, 2.61–5.14). Adjusting for sex, workplace, and time to first dose, ancillary HCWs (HR, 1.53; 95% CI, 1.03–2.28), medical HCWs (HR, 1.8; 95% CI, 1.18–2.74), and nursing HCWs (HR, 1.8; 95% CI, 1.18–2.37) received boosters earlier than administrative staff. No temporal relationship was observed for booster uptake, legislative changes, or COVID-19 case numbers. **Conclusions:** Vaccine hesitancy among HCWs had improved from first dose to booster, with timely booster vaccination among medical and nursing staff. Tailored education, risk messaging, and strategic legislation might help reduce delayed booster vaccination. This study was approved by the National Healthcare Group (NHG) Domain Specific Review Board (DSRB), Singapore on December 28, 2021 (Reg No. 2021/01120).

